# Searching for studies: A guide to information retrieval for Campbell systematic reviews

**DOI:** 10.1002/cl2.1433

**Published:** 2024-09-10

**Authors:** Heather MacDonald, Cozette Comer, Margaret Foster, Patrick R. Labelle, Scott Marsalis, Kate Nyhan, Zahra Premji, Morwenna Rogers, Ryan Splenda, Claire Stansfield, Sarah Young

**Affiliations:** ^1^ MacOdrum Library Carleton University Ottawa Ontario Canada; ^2^ University Libraries, Virginia Tech Blacksburg Virginia USA; ^3^ Medical Sciences Library Texas A&M University College Station Texas USA; ^4^ University of Ottawa, Library Ottawa Ontario Canada; ^5^ University Libraries, University of Minnesota‐Twin Cities Minneapolis Minnesota USA; ^6^ Cushing/Whitney Medical Library Yale University New Haven Connecticut USA; ^7^ Advanced Research Services University of Victoria Libraries Victoria British Columbia Canada; ^8^ NIHR ARC South West Peninsula (PenARC) University of Exeter Medical School Exeter UK; ^9^ Carnegie Mellon University Libraries Pittsburgh Pennsylvania USA; ^10^ EPPI Centre, UCL Social Research Institute, University College London London UK

**Keywords:** search, systematic reviews

## Abstract

This guide outlines general issues in searching for studies; describes the main sources of potential studies; and discusses how to plan the search process, design, and carry out search strategies, manage references found during the search process and document and report the search process.

## 1 ABOUT THIS GUIDE

1.1

This guide is derived from the information in Chapter 4 of The Cochrane Handbook (Higgins et al., 2023; Lefebvre et al., 2023). Carol Lefebvre, Eric Manheimer and Julie Glanville kindly gave permission to the original Campbell Collaboration (CC) Information Retrieval Guide authors to use the chapter and chapter updates as the basis for this guide. In 2015 (Kugley et al., 2017) and most recently in 2023 the Campbell Information Retrieval Methods Group (CC‐IRMG) revised this guide to reflect current CC areas of practice and recommendations in the Methodological Expectations of Campbell Collaboration Intervention Reviews (MECCIR), capture evolving practice and strategies for searching, and update links and descriptions of individual bibliographic and other resources.

This document outlines some general issues in searching for studies; describes the main sources of potential studies; and discusses how to plan the search process, design, and carry out search strategies, manage references found during the search process and document and report the search process. A list of abbreviations and definitions used in this guide can be found in Appendix V.

### 1.2 Who is this guide for?

This guide is meant for review authors and information specialists (IS) to reference in the planning and conduct of Campbell Systematic Reviews. The information in this guide is designed to assist authors wishing to undertake searches for studies and to provide background information so that they can better understand the search process.

This guide provides high‐level, overview information on information retrieval principles and is not a substitute for the Help sections of individual databases or thesauri. Researchers who wish to search particular sources should familiarise themselves with the database before beginning a search.

### 1.3 Citation

MacDonald, H., Comer, C., Forster, M., Labelle, P., Marsalis, S., Nyhan, K., Premji, Z., Rogers, M., Splenda, R., Stansfield, C., & Young, S. (2024). Searching for Studies: A Guide to Information Retrieval for Campbell Review. *Campbell Systematic Reviews,* Issue, [*e‐*page number]. [doi].

## 2 WORKING WITH AN IS/LIBRARIAN

### 2.1 Role of the IS/librarian

The fundamental premise of this guide is that information retrieval is an essential component of the systematic review, analogous to the data collection phase of a primary research study. A thorough and unbiased compilation of all potentially relevant studies is one of the key characteristics of a systematic review. If the literature located is unrepresentative of the population of completed studies, the remainder of the review process will be compromised (Lefebvre et al., 2023; Rothstein & Hopewell, 2009).

Librarians and IS are experts in searching. They can play an integral role in the production of Campbell reviews. There is increasing evidence to support the involvement of an IS in systematic reviews (Spencer & Eldredge, 2018; Wang & Lin, 2022) and evidence that IS involvement improves the quality of various aspects of the search process and reporting (Aamodt et al., 2019; Meert et al., 2016; Metzendorf, 2016; Pawliuk et al., 2024; Ramirez et al., 2022; Rethlefsen et al., 2015; Schellinger et al., 2021; Wang & Lin, 2022).

IS and librarians can offer support to authors in study identification from the early planning stage to the final write‐up of the review (Dalton, 2019; Foster, 2015; Ghezzi‐Kopel et al., 2022; Spencer & Eldredge, 2018). They may be able to provide training in, or if they are a member of the research team perform, some or all of the following:

- Selecting databases and other sources to search.
- Designing search strategies for the main bibliographic databases and/or trials registries.
- Running searches in databases and/or registries.
- Saving, collating, and sharing search results in appropriate formats.
- Documenting the search process in enough detail to be reproducible.
- Drafting the search methods sections of a Campbell Protocol and Review and/or Update.
- Ensuring that Campbell Protocols, Reviews and Updates meet the requirements set out in the MECCIR standards (https://doi.org/10.17605/OSF.IO/KCSPX) relating to searching activities for reviews.
- Obtaining full‐text documents for review teams when required.
- Providing advice and support on the use of reference management tools such as Zotero (https://www.zotero.org/) and EndNote (https://endnote.com/), and other software used in review production, including review management tools such as RevMan (https://revman.cochrane.org/info), Covidence (https://www.covidence.org/) and EPPI‐Reviewer (https://eppi.ioe.ac.uk/eppireviewer-web/home).

It is recommended that review authors seek guidance from an academic librarian or IS with experience in supporting systematic reviews. Researchers may want to consider inviting an IS or librarian to be part of their team to help ensure a robust search.

### 2.2 How to find an IS/librarian

Researchers at academic institutions can contact their institutional library to see if there is a librarian with experience in systematic reviews. Academic librarians may be able to offer support for systematic reviews or be part of a review team.

#### 2.2.1 What if you cannot find an IS/Librarian to collaborate with?

If you do not have access to a librarian, you can contact the Managing Editor of your Campbell Coordinating Group who can contact the IS peer reviewer for the Coordinating Group or the CC‐IRMG to see if there is a librarian interested in/willing to be part of a research group.

If no IS of librarian is available, you can participate in a variety of training opportunities including the CC online course on systematic review and meta‐analysis, or review the Campbell Training Resources (https://www.campbellcollaboration.org/research-resources/training-courses.html) or videos on searching in specific databases that can be found online.

Throughout the systematic review, collaboration is vital ‐ search development is no different. Work as a team to develop an exhaustive set of terms, design a logically sound search strategy with which one could reasonably expect to collect all available evidence related to your topic, scour the internet for relevant sources, documenting the process with enough detail that the search results could be reproduced.

### 2.3 Summary points

- If possible, invite an IS or librarian with experience in conducting searches for systematic reviews to collaborate as a co‐author on your review.
- If collaborating is not possible, consult with an IS or librarian prior to finalising your search strategy.
- If you are not sure how to access an IS or librarian, contact the Managing Editor of your CC Group.
- If you are unable to access an IS or librarian, refer to required conduct and reporting guidelines.
- Use existing training opportunities for systematic review methods and/or comprehensive searching.
- Familiarise yourself with the functional characteristics of the search interface for each of your databases and adjust your search strategy accordingly.

## 3 INFORMATION RETRIEVAL OVERVIEW

### 3.1 General issues

This document is meant to provide general guidance to reviewers and to establish minimum standards for key information retrieval tasks. Although the guide speaks specifically to individuals planning to conduct a Campbell review, the policies, procedures (White, 2009), and guidelines are applicable to anyone interested in implementing information retrieval methods that maximise coverage and minimise bias. For a more comprehensive discussion of information retrieval for systematic reviews see White's (2009) chapter entitled Scientific Communication and Literature Retrieval (White, 2009) and other reports that have described searching challenges for systematic reviews of various topics (Aromataris & Riitano, 2014; Beahler et al., 2000; Blackhall & Ker, 2008; Ogilvie, 2005; Wu et al., 2012).

The basic requirements of the systematic review search are outlined in methodological and reporting guidelines. Familiarising yourself with these guidelines early and often are vital to establishing a high‐quality search strategy for your review. For Campbell reviews, use the MECCIR standards (https://doi.org/10.17605/OSF.IO/KCSPX). The present document is also an important resource if developing a search strategy without an IS or librarian.

### 3.2 The nature of the social, behavioural, and educational sciences literature

Given the diverse nature of the research questions addressed in the social, behavioural, and educational sciences that encompass the CC's core areas of focus potentially relevant studies are likely to be widely distributed and unreliably categorised. While retrieval of information from the literature is a critical concern for any systematic reviewer, retrieval of information about complex social, behavioural, and educational interventions is likely to be especially challenging. Studies in the social sciences more often lack a structured abstract and strict adherence to common terminology compared to those of the medical sciences, resulting in the need for more sensitive, rather than specific, searches (Mallett et al., 2012; Petticrew, 2006, pp. 83–84). The review process also favours research produced in the Global North making finding studies published in the Global South challenging (Bol et al., 2023).

#### 3.2.1 Minimising bias

Systematic reviews of interventions require a thorough, objective, and reproducible search of a range of sources to identify as many relevant studies as possible (within resource limits). This is a key characteristic that distinguishes systematic reviews from traditional narrative reviews and helps to minimise bias and therefore increases the likelihood of producing reliable estimates of effects.

A search of one database alone is not considered adequate. A business‐related study investigating the effect of database choice on systematic reviews showed that each of the three databases searched provided one quarter of the unique search results found (Wanyama et al., 2022).

Going beyond the main subject database is important for ensuring that as many relevant studies as possible are identified and to minimise selection bias of those that are found (Lefebvre et al., 2023). In other words, relying exclusively on one database search will likely retrieve a set of studies unrepresentative of all studies that would have been identified through a comprehensive search of several sources. For example, ERIC (Education Resources Information Centre) is the main subject database for education research. However, many education topics and research questions may be informed by research in psychology (e.g., APA PsycInfo), sociology (e.g., Sociological Abstracts) or the health sciences (e.g., MEDLINE). Thus, searching across all of these databases, as well as in multi‐disciplinary databases like Scopus or the Web of Science citation indices, is necessary to be comprehensive.

Time and budget restraints require the review authors to balance the thoroughness of the search with efficiency in use of time and funds. The best way of achieving this balance is to be aware of, and try to minimise, the biases such as publication bias and language bias that can result from an inappropriately restrictive search.

### 3.3 Studies versus reports of studies

Systematic reviews typically treat original studies as the unit of analysis. Individual studies may be reported in multiple publications or be associated with other studies (e.g., post hoc analyses or surveillance studies). Related publications may report unique information or be a source of unwanted duplicate data. Every possible effort should be made to flag associated or related publications for inspection by the review authors.

### 3.4 Types of studies

This guide focuses on searching for studies that evaluate the effectiveness of interventions. Although the general guidance in this document should be applicable to any review, more targeted guidance may be important for reviews that target a different study type. For example for cost effectiveness reviews, authors may seek guidance on searching from Chapter 7 in Evidence‐Based Decisions and Economics (Glanville & Paisley, 2010) or methodologic reports (Alton et al., 2006; Glanville & Paisley, 2010; Glanville et al., 2009; McKinlay et al., 2006; Royle & Waugh, 2003; Sassi et al., 2002). When conducting an overview study or review of systematic reviews authors should consider other search outlets such as systematic review repositories and registries.

### 3.5 Copyright

It is Campbell policy that all review authors and others involved in the Collaboration should adhere to copyright legislation. With respect to searching for studies, this refers to adhering to the terms and conditions of use when searching databases and downloading records and adhering to copyright legislation when obtaining copies of articles. Review authors should seek guidance on this from their local academic librarian or copyright expert as copyright legislation varies across jurisdictions.

### 3.6 Summary points

- CC review authors should, if possible, seek collaboration or advice from an academic librarian or IS with experience conducting searches for systematic reviews.
- A search of one database alone is not considered adequate.
- To minimise bias during the information retrieval phase, search multiple databases.
- The unit of analysis in a systematic review is usually independent studies. Be aware that some studies are reported in multiple publications.
- CC policy requires that all review authors and others involved in the Collaboration adhere to database licensing terms and conditions of use and copyright legislation.

## 4 SOURCES TO SEARCH

### 4.1 Subject databases

Subject databases are generally the best way to identify an initial set of relevant reports of studies within a specific field. The majority of field specific databases, such as ERIC (education) and APA PsycInfo (behavioural and mental health), include abstracts, and may include links to the full text of the scholarly literature. Access to full‐text literature will vary based on open‐access status and subscriptions tied to a user's institutional affiliations. These databases usually index journal and non‐journal sources, as well as materials in languages beyond English. A key advantage of these databases is that they can be searched for keywords in the title or abstract and/or by using controlled vocabulary assigned to each record (see Section 6.4).

Decisions related to which subject‐specific databases are to be searched will be influenced by the topic of the review, access to specific databases, and budget considerations. In addition to field‐specific databases, there are also a variety of multi‐disciplinary databases (see Appendix I) which can be worthwhile to search.

Most of the subject‐specific databases are available on a subscription or ‘pay‐as‐you‐go’ basis, with a few databases being freely available. Databases may be available at no cost to the individual through national providers, site‐wide licences at institutions such as universities or hospitals, or through professional organisations as part of their membership packages. Access to databases is therefore likely to be limited to those databases that are available to members of the review team. Review authors should seek advice from their local librarian for access at their institution. For those review authors who do not have an affiliation with an institution or access to subscription databases, Appendix I offers a selection of free and low‐cost databases.

A selection of the main subject‐specific databases and multi‐disciplinary databases are listed in Appendix I.

### 4.2 General databases

#### 4.2.1 National and regional databases

Many countries and regions produce databases that concentrate on the literature produced in those regions, and which often include journals and other literature not indexed elsewhere. Access to many of these databases is available free of charge on the internet. Others are only available by subscription or on a pay‐as‐you‐go basis. Indexing complexity and consistency varies, as does the sophistication of the search interface, but they can be an important source of additional studies from journals not indexed in other international databases. It is important to note that some of these may not be available in English. Some examples are listed in Appendix I.

#### 4.2.2 Citation indexes

Citation indexes are bibliographic databases that record citations in addition to the usual article record. Examples include Web of Science and Scopus. These databases are multidisciplinary in nature and can be used for searching along with subject, national and regional databases.

Additionally, if relevant studies are difficult to find or not picked up by the database search strategy, they might still be retrieved by citation searching or citation chasing (Greenhalgh & Peacock, 2005). This is carried out by examining the included studies in related systematic reviews, by searching the references of identified relevant studies (backwards citation searching) or by searching for studies that cite relevant papers (forwards citation searching) (Hirt et al., 2024). Various studies have found this method complements the searches carried out in subject databases (Belter, 2016; Cooper, Booth, et al., 2018; Frandsen & Eriksen, 2023; Papaioannou et al., 2010). Google Scholar can also be used for forwards but not backwards citation searching. See Section 4.3.2 for more information about citation searching.

##### Web of Science

Web of Science, produced by Clarivate Analytics, is a platform comprising several databases. Its ‘Core Collection’ of databases includes Science Citation Index Expanded (SCIE), Social Science Citation Index (SSCI) and the Arts and Humanities Citation Index (AHCI) as well as two conference proceedings citation indexes. A given institution's ‘core collection’ may contain additional databases as well. These databases can be searched as a group or individually. Together these cover multiple disciplines including social science, business, the agricultural, biological, and environmental sciences, engineering, technology, applied science, medical and life sciences, and physical and chemical sciences.

Web of Science Core Collection contains over 91 million records from more than 22,000 journal titles, books, and conference proceedings, making it a valuable source of grey literature. It can be used for both forwards and backwards citation searching (Clarivate, 2024).

##### Scopus

Scopus, produced by Elsevier, includes content from more than 27,000 journal titles covering over 90 million records, including over 148 thousand conferences and 290 thousand books. Scopus subject coverage includes the social, physical, health and life sciences. It can be used for both forward and backward citation searching (Scopus, 2024).

#### 4.2.3 Full‐text journals available electronically

The full text of most journals is available electronically on a subscription basis or free of charge on the internet. In addition to providing a convenient method for retrieving the full text of articles of identified records that have been deemed relevant after title/abstract screening, full‐text journals can also be searched electronically similar to the way database records can be searched in a subject database. However, the search interface may have limited search and export functionality. In addition, it is recommended that searching of individual full text journals or publisher platforms (such as Wiley, ScienceDirect, etc.) be restricted to key journals that are not fully indexed in the databases being searched. This is because searching for keywords in full text can result in a highly imprecise search and unnecessary screening. Thesauri, if available, should be used in these databases to ensure any keyword searching is adapted for that resource. See the related Section 4.4 on Handsearching for more information about selecting key journals for individual searching.

Most academic institutions subscribe to a wide range of electronic journals, and these are therefore available free of charge to members of those institutions. Review authors should seek advice about electronic journal access from the library service at their local institution. Some professional organisations provide access to a range of journals as part of their membership package. In some countries similar arrangements exist through national licences. There are also several international initiatives to provide free or low‐cost online access to full‐text journals (and databases) over the internet, including the Health InterNetwork Access to Research Initiative (HINARI), the International Network for the Availability of Scientific Publications (INASP) and Electronic Information for Libraries (eIFL). Examples of some full‐text journal sources that are available free of charge without subscription are given in Appendix I.

It is recommended that a local electronic or print copy be maintained for any possibly relevant article found electronically in subscription journals, as the subscription to that journal may not be in perpetuity. The journal may cease publication or change publishers and access to previously available articles may cease. The same applies to journals available free of charge on the internet, as their availability might change in the future.

### 4.3 Other sources of study information

#### 4.3.1 Grey literature sources

While there are several types of grey literature, broadly, it is a body of information that may not be published in conventional sources such as books or journal articles. Grey literature sources include government and regulatory agencies, professional organisations, NGOs, industry, academic institutions, and so forth. Examples of grey literature include conference proceedings, theses/dissertations, white papers, and technical reports.

Conference abstracts and other grey literature have been shown to be sources of approximately 10% of the studies referenced in Cochrane reviews (Mallett et al., 2002). In a Cochrane methodology review, all five studies reviewed showed that published trials showed an overall greater treatment effect than grey literature trials (Hopewell et al., 2007). In a Campbell review on multisystemic therapy, the single largest and most rigorous experiment was not published in a peer‐reviewed journal or a book (Littell, 2005). Thus, failure to identify trials reported in conference proceedings and other grey literature might bias the results of a systematic review.

Some databases contain both published and unpublished literature. However, there are also a variety of resources available on the internet that provide access to grey literature. For a list of sources, see Appendix II of this guide; see also Rothstein and Hopewell (2009).

Grey literature searching can be time consuming. There are no set limits of how much is enough. The research team should discuss where and how to search for grey literature. Consideration should be given to the time and capacity required and explain the rationale for including sources.

##### Conference proceedings and meeting abstracts

More than one‐half of studies reported in conference abstracts never reach full publication, and those that are eventually published in full have been shown to be systematically different from those that are never published in full (Scherer et al., 2018). It is, therefore, important to try to identify possibly relevant studies reported in conference abstracts through specialist database sources or on the internet. Some databases such as Sociological Abstracts index conference and meeting abstracts. Conference and meeting abstract sources are listed in Appendix I.

##### Dissertations and theses

Dissertations and theses are often indexed in subject databases such as ERIC or APA PsycINFO, however there are also databases devoted to indexing this type of material and it is advisable to search these specific dissertation sources. See Appendix I for a selected list of some theses and dissertation databases.

#### 4.3.2 Existing review and publication reference lists

Some of the most convenient and obvious sources of references to potentially relevant studies are existing reviews. Reviews may also provide useful information about the search strategies used in their development. Copies of previously published reviews relevant to the topic of interest should be obtained and checked for references to the included (and excluded) studies.

Reviews may be found in The Campbell Library as well as The Cochrane Library, which includes the Cochrane Database of Systematic Reviews and the Cochrane Central Register of Controlled Trials (CENTRAL). Both databases provide information on published reviews. Several investigators have also published search methods for identifying systematic reviews in various areas (Avau et al., 2021; Boluyt et al., 2008; Bradley, 2014; DeLuca et al., 2008; Goossen et al., 2018; Honest et al., 2003; Rathbone et al., 2016; Wilczynski & Haynes, 2009; Wilczynski et al., 2007; Wong et al., 2006; Woodman et al., 2010). See Appendix I for other sources of existing systematic reviews.

Subject databases can also be used to identify review articles and guidelines. In APA PsycINFO, review articles may be indexed under the Methodology terms ‘Systematic Review’ and ‘Meta‐Analysis’ or under the Subject Heading ‘Literature Review’. In MEDLINE, reviews should be indexed under the Publication Type term ‘Meta‐analysis’ or ‘Review’.

Along with searching the references cited in existing systematic reviews and meta‐analyses, reference lists of identified studies may also be searched for additional studies (Greenhalgh & Peacock, 2005; Horsley et al., 2011). Citation searching may produce a different set of results compared to those produced from a keyword/controlled vocabulary search. However, since investigators may selectively cite studies with positive (or negative) results, searching reference lists is a supplementary information retrieval strategy only, and should be used as an adjunct to other search methods.

#### 4.3.3 Web searching

Web searching includes using internet search engines such as Google as well as searching websites of organisations relevant to the review topic. It is useful as a secondary resource to retrieve published studies not retrieved by other means, and can be a valuable source of grey literature (Coleman et al., 2020; Haddaway et al., 2015).

##### Search engines

Internet search engines such as Google Search and Google Scholar hold a huge amount of content and have basic search interfaces, which makes them difficult to search systematically (Stansfield et al., 2016). Therefore it is usually necessary to simplify searches, or run several searches using different combinations of key terms (Briscoe et al., 2020; Stansfield et al., 2016). Results are usually ranked according to an algorithm and may be influenced by the geographic location of the searcher (Cooper et al., 2021) and their search history (Google, 2024). It is usually necessary to restrict results to a predetermined number for screening (e.g., the first 100) or stop screening when the results become less relevant (Briscoe, 2018; Stansfield et al., 2016).

Search engines that have access to large, up‐to‐date corpuses of information include the following:

- Google (www.google.com)
- Microsoft Bing (www.bing.com)
- Yahoo! Search (search.yahoo.com)
- DuckDuckGo (https://duckduckgo.com/)

Both Google and Yahoo have additional country‐ and language‐specific versions, for example, www.google.ca and ca.yahoo.com/ for Canadian sites.

Google Scholar may also be used for web searching. Like Google Search, it is a search engine rather than a database. It indexes peer‐reviewed papers, theses, preprints, abstracts, and technical reports from all disciplines (Google Scholar, n.d.) by crawling the internet including institutional repositories, open access journals and preprint servers. Despite its searching and downloading limitations Google Scholar is a comprehensive search tool that can yield many articles regardless of organisational or institutional access. It is likely to return hits that have already been identified by the database searches, assuming these were carried out comprehensively (Bramer et al., 2013).

##### Websites

The websites to search will be determined by the review topic. Examples of potentially useful sites include content produced by Government departments, charities, or professional societies. See Appendix II for a list of some websites that provide access to this material.

Internet searching is usually carried out at the end of the search phase of a review to ensure that the most recent information is found. Review authors should record the website URL together with dates, search terms used, and any decisions regarding the number of results to screen (see Section Reporting additional search strategies). Relevant documents found should be downloaded or saved locally in case the link to the record is removed.

#### 4.3.4 Unpublished studies

Some completed studies are never published. Finding out about unpublished studies and including them in a systematic review when eligible and appropriate is important for minimising bias. There is no easy and reliable way to obtain information about studies that have been completed but never published.

Colleagues can be an important source of information about unpublished studies, and informal channels of communication can sometimes be the only means of identifying unpublished data. Formal letters of request for information can also be used to identify completed but unpublished studies. One way of doing this is to send a comprehensive list of relevant articles along with the inclusion criteria for the review to the first author of reports of included studies, asking if they know of any additional studies (published or unpublished) that might be relevant. It may also be desirable to send the same letter to experts or others with an interest in the area, either individually or through email lists or listservs. Open Science Framework (OSF) (https://osf.io/) can also be used to find unpublished studies.

It should be kept in mind that asking researchers for information about completed but never published studies has not always been found to be fruitful (Hetherington et al., 1989; Horton, 1997) though some researchers have reported that this is an important method for retrieving studies for systematic reviews (Greenhalgh & Peacock, 2005; Royle & Milne, 2003).

#### 4.3.5 Ongoing studies

It is also important to identify ongoing studies, so that when a review is later updated these can be assessed for possible inclusion. Awareness of the existence of a possibly relevant ongoing study might also affect decisions with respect to when to update a specific review. Unfortunately, no single, comprehensive, centralised register of ongoing trials exists (Manheimer, 2002). Efforts have, in the medical sciences, been made by several organisations to create databases of ongoing trials and in some cases trial results on completion, either on a national or international basis. Databases include Clinicaltrials.gov and the EU Clinical Trials Register (see Appendix I for list of Trial databases).

CC authors whose reviews concern or border on health‐related topics or outcomes, may find relevant studies in Social Care Online or the World Health Organisation (WHO) International Clinical Trials Registry Platform Search Portal from numerous international trial registers. OSF (https://osf.io/) may also contain ongoing studies including non‐health related studies.

To avoid unplanned duplication and enable comparison of reported review methods from other systematic reviews, authors may search PROSPERO (http://www.crd.york.ac.uk/PROSPERO), an international database of prospectively registered systematic reviews in health and social care.

#### 4.3.6 Institutional repositories

Institutional repositories are online resources that research institutions such as universities provide for collecting and disseminating intellectual output. Publication types may include journal articles, theses and dissertations, and often are a mixture of published and grey literature. These resources can be particularly useful for finding evidence produced in Global South countries (Mallett et al., 2012).

The Directory of Open Access Repositories (OpenDOAR) (https://v2.sherpa.ac.uk/opendoar/and the Register of Open Access Repositories (ROAR) (https://roar.eprints.org/) are comprehensive directories of academic open access repositories, providing both repository lists, as well as the possibility to search for repositories or search repository contents.

#### 4.3.7 Preprints

Preprint repositories are another potential avenue to search for recent studies. A preprint is a research paper posted on a public server that has not undergone formal peer review. Their inclusion in systematic reviews can be controversial as there is tension between wanting to include relevant unpublished data and ensuring the study meets quality criteria for publication and does not change post publication (Brietzke et al., 2023).

### 4.4 Handsearching key journals

Handsearching traditionally involves a manual page‐by‐page examination of the entire contents of a journal issue or conference proceedings to identify eligible studies. Electronic journals and online conference proceedings can also be ‘handsearched’, by scrolling through contents online. Like other supplementary search methods, handsearching might locate studies not indexed in bibliographic databases or that were missed by the database searches.

A Cochrane Methodology Review found that a combination of handsearching and electronic searching was necessary for full identification of relevant reports published in journals, even for those that are indexed in MEDLINE (Hopewell et al., 2007). A study in 2008 found that handsearching was useful for identifying trials reported in letters or abstracts (Richards, 2008). More recently, Cooper, Varley‐Campbell, et al. (2018) found that handsearching had the best recall compared with other methods, but efficiency was poor.

As handsearching is a labour‐intensive process, reviewers might consider prioritising journals in which significant numbers of included studies have been found through other search methods. This will ensure picking up studies that have not yet been indexed by the databases.

### 4.5 A note about predatory journals

Predatory journals are journals that claim to be legitimate research publications but misrepresent their publishing and editorial practices (Elmore & Weston, 2020). The quality of articles included in these types of journals can be questionable (Moher et al., 2017). Unfortunately, they can be found in some bibliographic databases (Boulos et al., 2022; Dadkhah et al., 2017) meaning they can end up in database search results. Section 6.5.6 provides some guidance on dealing with predatory journals.

### 4.6 Summary points

As there is no preferred single source of studies or trials that can be searched for Campbell reviews, a broad selection of databases and other sources needs to be searched to ensure a comprehensive search strategy.

- Consult a librarian or IS to select national, regional, and subject‐specific databases that index literature related to the topic of the review.
- Consider alternate sources of information including conference abstracts, technical reports, theses and dissertations, and grey literature sources to complement the database search retrieval.
- Search trial registries to identify ongoing studies for possible inclusion in the review.
- Review reference lists of existing reviews and included studies for additional studies.
- Contact experts in the field to identify additional studies, and unpublished or ongoing research.
- Handsearch the tables of contents of key journals to identify newly published/non‐indexed publications.

## 5 PLANNING THE SEARCH

### 5.1 The CC review process

Before submitting a Campbell review, reviewers must first submit a title registration form that outlines the intended scope of the review. Once accepted, authors will submit a protocol to their CC group. Referees will review this protocol to ensure that all the necessary steps are to be completed correctly. This evaluation includes an extensive review of the search process used to identify studies. An information retrieval checklist (see Appendix IV) is used both at this stage and at the final review stage in the peer review process. It is recommended that reviewers consult this checklist to ensure their protocol matches the criteria that will be used to judge completeness.

### 5.2 Seed articles and search validation

Seed articles, also referred to as benchmark studies, are those articles that are known to meet the eligibility criteria of the review. They may be known to the review team at the outset or may be gathered via exploratory searching, citation harvesting from previous reviews on a similar topic, or contacting experts in the field. Each seed article should be evaluated against the eligibility criteria by the review team to ensure they meet criteria for inclusion and would pass, at a minimum, the title and abstract screening. However, seed articles that only partially meet the inclusion criteria may sometimes be included, especially where a search concept is known to be complex. In this case, the usefulness of these seed articles may be limited to only the relevant search concepts. Establishing a seed article set should be done as a precursor to the creation of a search strategy. The size of the seed article set depends on the breadth of the scope of the review. To be useful, it should be representative of the diversity of evidence expected to be included in the review This can include, but is not limited to, discipline, geographic region of the study, timeframe of publication, and variables pertaining to the review topic (such as study designs, independent and dependent variables).

The seed article set can be used during development of the search strategy and is also used as a test list for checking the performance of the search strategy. During search development, the seed set can be harvested for terms found in the title, abstract, and author‐keyword fields, and for controlled vocabulary terms applied to these records in specific databases. After creation of the search, the seed articles can be tested for retrieval against the search strategy, to validate search logic, and characterise the performance of the search strategy. Ideally the test set should be retrieved in its entirety across the combined database search results. Reporting of the validation process used and the articles included in the test set is recommended either at the protocol stage or in the final review manuscript (Page, Moher, et al., 2021; Pullin et al., 2022).

### 5.3 Search updates

#### 5.3.1 In‐process review search updates

Searches for in‐process reviews may need to be updated before submitting for publication. Cochrane's MECIR specifies that searches should be updated if more than 12 months have passed, although 6 months is preferred. Several approaches may be employed for updating the searches. Where available, ‘alerts’ may be set up to automatically run the search at set intervals and new matching records be screened for inclusion in the review. Maintaining Search Summary Tables can help streamline search updates (Rogers et al., 2024). Searches may also be manually updated and either deduplicated against the existing screened record set or filtered against the record load date. Manual updating of searches is especially advisable for subject databases, such as MEDLINE and APA PsycInfo, due to annual thesaurus updates or changes to search syntax.

#### 5.3.2 Updating published reviews

When a published Campbell review is updated, the search process (i.e., deciding which databases and other sources to search and for which years) will have to be reviewed. Those databases that were previously searched and are considered relevant for the update will need to be searched again.

The previous search strategies will need to be updated including checking for changes to controlled vocabulary terms or changes in search syntax. If any of the databases originally searched are not to be searched for the update this should be explained and justified. New databases or other sources may have been produced or become available to the review authors or IS and these should also be considered.

**TIP**: Search strategies within a database may be saved for future use but the strategies may have to be adjusted due to new‐found keywords or changes in the database provider's search software.

It is also recommended to check included studies against Retraction Watch (https://retractionwatch.com/) to ensure studies have not been retracted. For more information about how to deal with retracted publications see the Cochrane Handbook Technical Supplement to Chapter 4, Section 3.9 Identifying fraudulent studies, other retracted publications, errata and comments: further considerations (Lefebvre et al., 2023).

Consult Sections 9.2 and 9.3 for further information on the specific details to document and report in the final review manuscript.

### 5.4 Summary points

- Allocate sufficient time to plan search strategies and tailor strategies for selected databases.
- A diverse set of seed articles identified at the early stages of the review can be useful for designing, testing, and validating the search strategy. The seed article set and its use should be detailed in the review methods section of the protocol and/or review manuscript.
- When updating searches, original strategies may have to be adjusted due to changes in the database interface, newly found keywords, or changes in controlled vocabulary.

## 6 DESIGNING SEARCH STRATEGIES

### 6.1 An introduction

This section highlights some of the issues to consider when designing search strategies for databases within the social sciences, but it does not completely address the many complexities in this area. There are two important reasons for this:

- Range of databases: Given the multidisciplinary nature of most social science research questions and the large selection of social science related databases, searches must be implemented in multiple databases. Terminology (both keywords and controlled vocabulary) will vary across these databases as different disciplines often use different words to describe the same thing.
- Database providers: The same database can be supplied by different organisations, called database providers or platforms. Examples of database providers are EBSCO, Gale, Clarivate, Ovid, and ProQuest. For example, ERIC is a database supplied by EBSCO, ProQuest, and Ovid. Each database provider designs their own search software and packages the data within the database differently (e.g., some fields may be included, others may not). This means that commands, operators, limiting options, and availability of fields will differ, resulting in the need to understand each provider's software thoroughly.

Given the above, customised search strategies must be constructed for each database as terminology will vary across disciplines, and the way one searches will differ across databases. It is for these reasons that the construction and implementation of searches requires the skills of an IS or academic librarian as one risks missing retrieving key studies if searches are poorly constructed or improperly implemented.

The review inclusion criteria will inform how the search is designed. The inclusion criteria may specify the eligible study designs, participants, interventions, and outcomes. Other aspects to consider in planning a search include:

- Geographic considerations
- Publication language
- Publication dates (keeping in mind that retrieval tools have different beginning dates and may not index very old material)
- Relevance of data from unpublished sources
- Type of resource and study design

### 6.2 Sensitivity versus precision

Searches for systematic reviews aim to be as extensive as possible to ensure that as many as possible relevant studies are included in the review. It is, however, necessary to strike a balance between striving for comprehensiveness and maintaining relevance when developing a search strategy. Increasing the comprehensiveness (or sensitivity) of a search will reduce its precision and will retrieve more non‐relevant articles.

Sensitivity is defined as the number of relevant reports identified divided by the total number of relevant reports in existence. Precision is defined as the number of relevant reports identified divided by the total number of reports identified. Developing a comprehensive search is an iterative process in which the terms that are initially used may be modified based on what has already been retrieved.

### 6.3 Search strategy structure and components

The structure of a search strategy should be based on the main concepts being examined in a review. For a Campbell review, the review title and stated objectives should provide these concepts. The eligibility criteria for studies to be included will further assist in the selection of appropriate subject headings and keywords for the search strategy. It is usually unnecessary and even undesirable, to search on every aspect of the review's research question. Although a question may address particular settings or outcomes, these concepts may not be well described in the title or abstract of an article and are often not well indexed with controlled vocabulary terms.

Generally speaking, a search strategy to identify intervention studies will typically include two concepts: (1) the condition of interest, that is, the population and (2) the intervention(s) evaluated. Sometimes a third concept may be included, the outcome(s), although as mentioned not all outcomes may be mentioned in the title or abstract. Search filters or limiting commands may be used to further narrow the results by study design, document type, dates, and so forth (see Section 6.5.6 Search filters vs. limiting commands).

Once decisions have been made regarding which databases will be searched, the following key decisions will need to be made:

- What are the key concepts to be searched?
- How are these key concepts represented in each relevant (or related) field and across different cultures?
- What are the related terms for these key concepts?
- How are these key concepts represented in the controlled vocabulary within each database?

For systematic reviews, a single search strategy is designed to capture all relevant literature. Troubleshooting the search strategy is usually done in the main database(s) expected to provide the majority of relevant results.

### 6.4 Keywords and controlled vocabulary

Many databases can be searched in two ways. The first is to use keywords, or words which are found in database fields such as the title or abstract field (sometimes called ‘free text searching’ or ‘natural language terms’). The second is based on subject headings that are assigned to individual records when they are entered into the database. These terms may be described as ‘subject headings’, ‘thesaurus terms’, or ‘controlled vocabulary’. For systematic reviews it is recommended that both approaches are used in combination.

#### 6.4.1 Identifying relevant controlled vocabulary

Databases often have their own thesaurus of controlled vocabulary terms, for example, ERIC uses the ERIC Thesaurus, PsycINFO uses APA Thesaurus of Psychological Index Terms, and Medline uses MeSH (Medical Subject Headings). These thesauri are organised into hierarchies of headings and subheadings that can be added into database search strategies by selecting them. Controlled vocabulary is useful because it uses words to describe concepts that might not be represented by keywords in the title or abstract fields. It is important to note that the controlled vocabulary is generally different between databases; hence a subject heading in ERIC may not have an equivalent in APA PsycINFO and vice versa.

If a subject heading includes a number of subheadings, it can be ‘exploded’. This means that the main heading together with all the narrower headings will be searched. It is important to check that all subheadings are relevant to the review question, if not, specific subheadings can be selected. Headings can also be selected as being a Major Topic (called ‘Focus’ in Ovid), which is where an indexer has deemed the concept to be a key one for that record. It should be noted that using this function may compromise the sensitivity of the search, and thus is rarely used in systematic review searches.

1 **TIP**: Each database has its own controlled vocabulary, which is listed in a database's thesaurus. When planning a search, it is useful to scan the thesaurus to get a sense of the terminology used, and view broader, narrower, and related subject terms.

Most interfaces offer an option to browse the hierarchy of subject headings associated with a specific database. The list of subject headings will also include a search function, so keywords can be searched for within the hierarchy and mapped against the relevant heading. Usually, subject headings come with a scope note that provides a definition for the term. Headings can then be selected and added to the search. Subject headings may have a list of similar or related subject headings. These can be selected and used in the search strategy if they are relevant to a particular concept.

Before beginning the search, it is good practice to identify reviews that include similar concepts and examine their search strategies to see what controlled vocabulary they used. Similarly, records of studies that meet the inclusion criteria (i.e., seed articles, see Section 5.2) can be examined within the database. The list of subject headings and author assigned keywords should be available within the database record. Online tools such as the Yale MeSH Analyzer (https://mesh.med.yale.edu/) and PubReminer (https://hgserver2.amc.nl/cgi-bin/miner/miner2.cgi) can help with this process.

Many databases offer useful features such as ‘Related Searches’ or ‘Find Similar Results’, or a list of subject headings extracted from the retrieved set will be displayed in a sidebar, along with the number of hits retrieved for each heading. The former is database specific and can be used to find related content and search terms based on the database indexing. The latter feature is especially useful as it may introduce new subject headings that were not previously considered while also providing an indication of the number of records within the database that contains a particular subject heading.

#### 6.4.2 Identifying relevant keywords

Keywords can be identified through examining the titles and abstracts of relevant studies. It is essential that for any word selected, related terms and synonyms are also identified by using a thesaurus or dictionary. For example, for the concept of parental involvement the following terms could be considered: parental support, family support, family participation, and so forth. Review teams usually include an expert in the field who can also help identify synonyms that might have been overlooked. Checking search strategies in related systematic reviews is also a useful way of identifying more search terms.

It is important to remember that English words might be spelt differently across geographical regions, for example, color (US) and colour (UK). Both spellings should be used in a search strategy. Similarly, some words for the same thing vary by country. This is particularly true of drug names, for example, acetaminophen (US) and paracetamol (UK).

Reviewers should consider the individual database to be searched before choosing keywords. Databases which are topic‐specific, for example, ERIC, APA PsycINFO or Ageline are useful because they are already limited in scope. It should not be necessary therefore to search for the term ‘education’ in ERIC, or ‘Older adults’ in Ageline or ‘Psychology’ in APA PsycINFO. Including these terms with other concepts may be overly limiting and will not be useful in databases that are already focused on these topics.

#### 6.4.3 Text mining for term selection

Text mining is a technique that uses natural language processing, machine learning or statistical techniques (like term frequency analysis) to transform human language into something that a machine can interpret and analyse (Elliot et al., n.d.). Text mining can be used to assist in the identification of relevant text words for search strategy development and to improve the sensitivity and precision of searches. It should not completely replace the work of domain experts and the IS in identifying keywords but can be used to find keywords missed by the traditional term harvesting techniques described above and to generate a starting point for search strategy development.

Text mining in the context of term selection for systematic reviews typically involves the use of a set of relevant studies. These can be identified using targeted searches of a database. It can be useful to analyse text from relevant references that are not findable from free‐text terms, such as a set of seed or benchmark studies identified by the research team, to inform discovery of additional search terms. Natural language processing or other text mining techniques are then applied to the titles, abstracts and/or full text of these studies. This should begin with the removal of ‘stop words’ (common words not likely to be meaningful keywords, like ‘and’, ‘or’ and ‘the’), and techniques to identify important terms such as term frequency analysis or keyword co‐occurrence. Once identified, these keywords can be screened, and authors and IS can make informed decisions about a word's relevance to the search.

There are many tools and software available to facilitate the application of text mining for search strategy development, some of which are general text mining tools and some specifically developed for evidence synthesis. For more information about the use of text mining in systematic reviews and associated tools, see Section 3.2.3 of the Technical Supplement of Chapter 4 of the Cochrane Handbook (Lefebvre et al., 2023). However, some of these tools are only developed for use with references from healthcare databases, such as PubMed, whereas generic tools can be useful for combining references obtained from different sources. Lefebvre et al. (2023) discusses some of the ways text mining could be used for informing searches.

There is growing interest in the use of artificial intelligence tools such as ChatGPT to assist in systematic review processes. The value of these tools is just emerging and the evidence of their efficacy in designing a comprehensive search strategy is unclear (Alshami et al., 2023). In the meantime, ChatGPT or similar tools should be used cautiously.

### 6.5 Formulating search strategies

Some key decisions must be made when formulating search strategies. These can include considering how best to represent concepts using both subject headings or controlled vocabulary alongside keywords or natural language terms. In addition, decisions need to be made around the use of truncation, Boolean operators, proximity searching, nesting, and field searching (keeping in mind that each database/search interface has a unique syntax). Finally, several limit options must be examined to determine their appropriateness within the context of conducting systematic reviews. Thought should be given to how useful or necessary it may be to limit by document type, by year of publication, or by study design.

#### 6.5.1 Boolean operators (AND, OR and NOT)

A search strategy should build up the controlled vocabulary terms, keywords, synonyms, and related terms for each concept, joining together the terms within each concept with the Boolean ‘OR’ operator (see example search strategies in Section 6.5.8). This means that retrieved articles will contain at least one of these search terms. Search concepts should be developed for the population (or condition) and intervention(s). These should then be joined together with the ‘AND’ operator. When using multiple operators, it is important to nest the search terms. Nesting refers to the use of parentheses to organise a search statement (see Section 6.5.8).

This final step limits the retrieved set to articles that address all included concepts. A note of caution about this approach is warranted however: if an article does not contain at least one term from each of the three sets, it will not be retrieved. For example, if an index term has not been added to the record for the intervention and the intervention is not mentioned in the title and abstract, the article would be missed. A possible remedy is to omit one of the concepts and decide which records to check based on the number retrieved and the time available to check them.

The ‘NOT’ operator should be avoided where possible to avoid inadvertently removing relevant records from the search set. For example, searching for records indexed as (female NOT male) would remove any record that was about both males and females.

#### 6.5.2 Phrase searching and proximity operators

Most databases offer the ability to search for two or more words, in the order specified, commonly by enclosing keywords within quotes. An example of phrase searching would be: ‘parental involvement’.

Many database providers allow the searcher to use proximity operators (e.g., NEAR, WITHIN, adj), sometimes called adjacency operators, that specifies the relationship of two or more terms within a field. This results in higher sensitivity than simple phrase searching but greater precision than use of the ‘AND’ operator. It is, therefore, desirable to use this operator where available and relevant.

For example, the proximity operator in EBSCO databases (N#) will find the search terms within a certain number of words of each other regardless of their order. So (parental N3 involvement) will find these two keywords within three words of each other regardless of the order in which they appear. Similarly, the proximity operator (W#) finds the words if they are within a certain number of words of one another, but in the order in which they have been entered. So (parent* W3 involvement) will find ‘parental involvement’ but not ‘involvement of the parents’.

The availability and commands of proximity operators will vary depending on the provider of the database. It is therefore important to consult the Help or Search Tips section within each database.

#### 6.5.3 Truncation and wildcards

To be as comprehensive as possible, it is important to include a wide range of keywords for each of the concepts being searched, including relevant synonyms, related terms, and variant spellings. This might include the use of truncation and wildcards. Truncation, also called stemming, is a technique that broadens your search to include various word endings. The asterisk (*) symbol is commonly used for truncation, but some databases may use other symbols. For example, parent* would retrieve results containing the terms parent, parental, parenting, and so forth. Wildcards are used to represent one or more characters. Wildcards are commonly used to find words with variable spellings. For example, colo?r could retrieve results containing the terms color or colour. Refer to the help guide for each database to check the proper truncation and wildcard symbols to use in your search.

#### 6.5.4 Field searching

Field searching involves searching specific database fields such as the title, abstract, or journal name. Field searching can be used instead of the default search or a combined field search in a database. For example, the Topic field in Web of Science includes title, abstract and author keywords. In CINAHL the default search includes Title, Abstract, and Subject Headings. Limiting to title, abstract and keyword fields is a more specific search and can reduce the number of results found. If your search terms are exhaustive within each concept, and those terms are not present in the title, abstract, or keywords, then it is unlikely that the article would be relevant to the scope of the review.

#### 6.5.5 Language, date and document format restrictions

Review authors should justify the use of any restriction in the search strategy. Whenever possible, review authors should attempt to identify and assess for eligibility all possibly relevant reports irrespective of language of publication. However, translation of included studies at the data extraction stage can be time consuming and/or costly. Ideally no language restrictions should be included in the search strategy to minimise bias. The review team can use free online translation tools to translate titles and abstracts to determine eligibility for screening.

The application of a publication date restriction will depend on the research question being addressed. For example, if it is known that relevant studies would have been conducted only after a specific date (e.g., web‐based learning in schools would not be addressed prior to the mid‐1990s), a justifiable use of publication date limits can be applied during the search.

Any information about an eligible study may contain valuable details for analysis. Letters, comments, errata and preprints may all contain relevant information about a study (Iansavichene et al., 2008; Oikonomidi et al., 2020; Zeraatkar et al., 2022). However, some databases contain news and wire feeds which review teams may not wish to include in their search. Careful consideration should be given to document format restrictions.

Review teams should decide how they will deal with predatory journals. Identifying predatory journals at the search stage can be difficult as they can be found in bibliographic databases (Boulos et al., 2022; Dadkhah et al., 2017). Using a critical appraisal tool may help reduce the risk of including poor quality studies (Ross‐White et al., 2019). Rice et al. (2021) suggest checking if open access journals are listed in the directory of open access journals (DOAJ) (https://doaj.org/) or conducting a sensitivity analysis with predatory papers excluded from the synthesis. Munn et al. (2021) provide guidance on dealing with predatory journals including checking if journals adhere to Committee on Publication Ethics (COPE) (https://publicationethics.org/) core practices, using the Think, Check, Submit checklist (https://thinkchecksubmit.org/) to determine journal credibility, or checking against a list of predatory journals.

#### 6.5.6 Search filters versus limiting commands

Search filters, also called ‘search hedges’, are predefined search strategies that are designed to retrieve specific types of records, such as those of a particular methodological design, geographic location or topic. Limiting commands, on the other hand, are built‐in database‐specific filters that can be applied once a set of results has been generated or when the search is executed, depending on the database and platform. For example, in Web of Science, there is a Categories limiting command based on Web of Science journal subject groups, and ERIC has a Document type limiting command for different types of publications.

Used extensively in the medical and health sciences, search filters may also be used when searching in the social sciences, but with some caution. A search filters web site has been developed by the UK InterTASC Information Specialists Subgroup (ISSG), which is the group of information professionals supporting research groups within England and Scotland providing technology assessments to the National Institute for Health and Clinical Excellence (NICE) (Glanville et al., 2008). The purpose of the website is to list search filters and to point to critical appraisals of the various filters. The site includes, amongst others, methodological filters for identifying systematic reviews, randomised and non‐randomised studies and qualitative research in a range of databases and across a range of service providers (https://sites.google.com/a/york.ac.uk/issg-search-filters-resource/home).

However, there are two main challenges with using search filters in the social sciences. Firstly, databases in the social sciences tend not to be as thoroughly indexed as those in medicine and may use indexing inconsistently, if at all. Similarly, if a search filter that uses keywords is used, potentially relevant studies may be missed. This is especially true for methodology as it is often not found in social sciences abstracts. This may call for a broader approach to searching for methodological contents. Searching for specific study types along with general terms might be more useful.

1 **TIP**: When searching for methodological content include specific study types and general terms rather than relying solely on search filters.

Searchers are discouraged from using database limiting commands when conducting systematic review searches as it is not always clear how these filters are designed and how they are applied to generate a set of results. Rather, a search filter or concept should be incorporated in the search. One exception is publication date, which can be reliably applied using a database's limiting command feature. However, the use of any search filter or limiting command should be documented and justified.

#### 6.5.7 Adapting search strategies across databases

Once a complete search strategy is designed, tested, and finalised for the main database, a similar strategy is used for the other databases to be searched. The search will need to be customised according to the controlled vocabulary and syntax of each database and database platform. Note that in some databases, certain important concepts may also be represented in fields other than those for controlled vocabulary index terms, and natural language terms in titles or abstracts. For example, Classification Code or Age Group may contain relevant concepts that can be used in a search strategy. Tools exist that can help with adapting or translating a search strategy such as Polyglot (https://polyglot.sr-accelerator.com/), Medline Transpose (https://medlinetranspose.github.io/) and the Cochrane Database Syntax guide (EPOC, 2017).

Navigating differences in functionality and syntax across database interfaces can be challenging. Most interfaces for academic databases provide a search guidance handbook that highlights search functionality (e.g., search field code definitions, adjacency/proximity operators, exact search options). They also often offer online training via videos and written guidance. Although there will be commonalities (e.g., functionality of Boolean operators), it is important to be aware of subtle changes so that you can adjust your search strategy to accommodate the search interfaces for each database.

#### 6.5.8 Example

Boxes 6.1 and 6.2 provides examples of a search strategy in two databases on the topic of parental involvement and academic performance in elementary school children. Note that a combination of controlled vocabulary (indicated by MAINSUBJECT.EXACT or DE) and keywords (in the Title or TI or Abstract or AB fields) were used, along with Boolean and proximity operators, phrase searching, truncation, and date limiters. In the examples, you can see that the database platforms (ProQuest and EBSCO) provide a line‐by‐line approach where search strings for individual concepts are entered and then combined into a single search using the line numbers associated with each concept block.

Box 6.1 **Searching within ERIC (ProQuest platform).**

1

**Table:** 

Set#	Searched for	Annotations
S1	MAINSUBJECT.EXACT(‘Elementary School Students’) ORMAINSUBJECT.EXACT(‘Elementary Education’) ORMAINSUBJECT.EXACT(‘Kindergarten’) ORMAINSUBJECT.EXACT(‘Primary Education’)	Concept 1 (population) controlled vocabulary terms
S2	abstract(((elementary OR primary) NEAR/2 school*) OR kindergarten*) OR title(((elementary OR primary) NEAR/2 school*) OR kindergarten*)	Concept 1 keyword terms (synonyms) searched in abstract and title fields
S3	[S1] OR [S2]	Combine concept 1 terms
S4	MAINSUBJECT.EXACT(‘Parent Participation’) ORMAINSUBJECT.EXACT(‘Parent School Relationship’) ORMAINSUBJECT.EXACT(‘Parent Teacher Conferences’) ORMAINSUBJECT.EXACT(‘Parents as Teachers’) ORMAINSUBJECT.EXACT(‘Parent Role’) ORMAINSUBJECT.EXACT(‘Family Involvement’)	Concept 2 (intervention) controlled vocabulary terms
S5	abstract(((Parent* or family or families) NEAR/2 involvement) OR ((Parent* or family or families) NEAR/2 support) OR ((Parent* or family or families) NEAR/2 participation)) OR title(((Parent* or family or families) NEAR/2 involvement) OR ((Parent* or family or families) NEAR/2 support) OR ((Parent* or family or families) NEAR/2 participation))	Concept 2 keyword terms (synonyms) searched in abstract and title fields
S6	[S4] OR [S5]	Combine concept 2 terms
S7	MAINSUBJECT.EXACT(‘Science Achievement’) ORMAINSUBJECT.EXACT(‘Reading Achievement’) ORMAINSUBJECT.EXACT(‘Academic Achievement’) ORMAINSUBJECT.EXACT(‘Writing Achievement’)	Concept 3 (outcome) controlled vocabulary terms
S8	abstract(‘academic achievement’ OR ‘academic performance’) ORtitle(‘academic achievement’ OR ‘academic performance’)	Concept 3 keyword terms (synonyms) searched in abstract and title fields
S9	[S7] OR [S8]	Combine concept 3 terms
S10	[S3] AND [S6] AND [S9]	Combine concepts
S11	([S3] AND [S6] AND [S9]) AND yr(2000–2024)	Limit by date

Box 6.2 **Searching within Child Development & Adolescent Studies (EBSCOhost platform).**

1

**Table:** 

#	Query	Limiters	Annotations
S11	S3 AND S6 AND S9	Publication Date: 20000101‐20221231	Limit by date
S10	S3 AND S6 AND S9		Combine concepts
S9	S7 OR S8		Combine concept 3 terms
S8	AB (‘academic achievement’ OR ‘academic performance’) OR TI (‘academic achievement’ OR ‘academic performance’)		Concept 3 keyword terms (synonyms) searched in abstract and title fields
S7	(DE ‘academic achievement’)		Concept 3 (outcome) controlled vocabulary terms
S6	S4 OR S5		Combine concept 2 terms
S5	AB (((Parent* or family or families) NEAR/2 involvement) OR ((Parent* or family or families) NEAR/2 support) OR ((Parent* or family or families) NEAR/2 participation)) OR TI (((Parent* or family or families) NEAR/2 involvement) OR ((Parent* or family or families) NEAR/2 support) OR ((Parent* or family or families) NEAR/2 participation))		Concept 2 keyword terms (synonyms) searched in abstract and title fields
S4	(DE ‘parent participation in education’) OR (DE ‘parent participation in elementary education’) OR (DE ‘parent participation in kindergarten’) OR (DE ‘parent participation in preschool education’) OR (DE ‘parent participation in primary education’)		Concept 2 (intervention) controlled vocabulary terms
S3	S1 OR S2		Combine concept 1 terms
S2	AB (((elementary OR primary) N2 school*) OR kindergarten*) OR TI (((elementary OR primary) N2 school*) OR kindergarten*)		Concept 1 keyword terms (synonyms) searched in abstract and title fields
S1	(DE ‘kindergarten’) OR (DE ‘elementary schools’) OR (DE ‘elementary education’) OR (DE ‘primary education’)		Concept 1 (population) controlled vocabulary terms

### 6.6 Supplementary search techniques

Additional search methods are often employed to increase comprehensiveness and thus minimise bias of searches, especially for reviews that require qualitative evidence (Frandsen & Eriksen, 2023).

Contacting experts, as mentioned earlier, can help identify unpublished or ongoing studies (Sections 4.3.4 and 4.3.5). Experts can also help with identifying potentially relevant seed articles by leveraging their knowledge of and expertise in the field.

Handsearching, or manually reviewing all the content from a journal, conference proceeding, and so forth (Section 4.5), is recommended particularly for finding observational research and topics without standardised terminology (Kwon et al., 2014). Paperfetcher (https://paperfetcher.github.io/) is a tool that facilitates handsearching and citation searching for systematic reviews.

Checking the references of included studies (Section 4.3.2) is an important additional search step to perform. Citation searching, also called citation tracking, tracing, chasing, chaining, or snowballing (Section 4.2.2), has been particularly useful for reducing the risk of missing available, relevant material (Frandsen & Eriksen, 2023; Hirt et al., 2023). For systematic review topics that are difficult to search, both backward and forward citation searching should be considered (Hirt et al., 2024). Citationchaser (https://www.eshackathon.org/software/citationchaser.html), SpiderCite (https://sr-accelerator.com/#/spidercite) and Paperfetcher are tools to help with forward and backward citation searching.

### 6.7 Search strategies for internet search engines

Many of these search strategies may also be applied in internet search engines (Google, Google Scholar, Microsoft Bing, etc.). For example, phrase searching, Boolean operators and limiting features are typically all offered. Using the search engine's Advanced search screen can provide an easy way of accessing these features.

Boolean logic can be used by entering keywords into the following search windows:

- all these words (AND),
- this exact word or phrase (Phrase searching),
- any of these words (OR), and
- none of these words (NOT).

If keywords are entered into multiple search windows, the system will AND each search statement. For example, the search strategy entered into Google's Advanced search screen illustrated in Box 6.3, may be translated as: elementary AND (performance OR achievement) AND ‘parental involvement’.

1 **TIP**: Using the File type restriction is an effective way to limit your results to reports in Word or PDF documents.

Box 6.3 **An example of Google's Advanced search screen.**

1

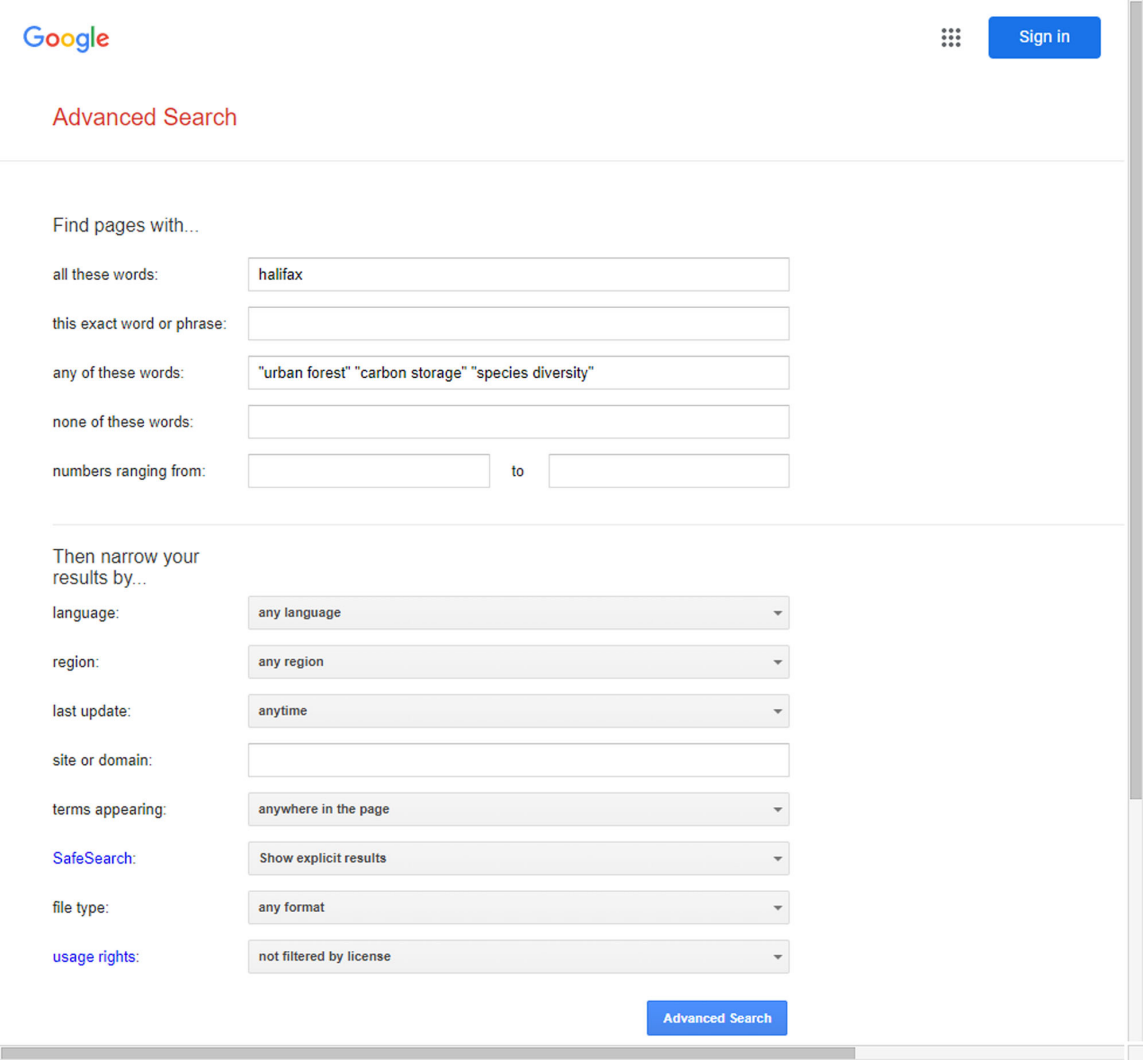

Similarly, search results may be narrowed by using specific limits such as: Language, Date, File type, or Domain.

1 **TIP**: Strategies should be precise to reduce retrieving a large number of records. Keywords such as ‘study’ or ‘studies’ or ‘control group’ may be used to limit the results to empirical research.

### 6.8 Peer review of search strategies

As recommended throughout this document and in other systematic review standards and guidelines, an IS or librarian with evidence synthesis expertise should be involved in developing the search strategy. It is also recommended that the search strategy go through an additional process of peer review prior to protocol or manuscript submission. Peer review of search strategies, as distinct from peer review of the whole systematic review, is increasingly recognised as a necessary step in designing and executing high‐quality searches to identify studies for possible inclusion in systematic reviews, in addition to IS involvement (Folb et al., 2020; Neilson, 2021). Studies have shown that errors occur in the search strategies underpinning systematic reviews and that search strategies are not always conducted or reported to a high standard (Layton, 2017; Mullins et al., 2014; Ramirez et al., 2022; Salvador‐Olivan et al., 2019; Sampson & McGowan, 2006). The Preferred Reporting Items for Systematic Reviews and Meta‐Analyses Extension for Searching (PRISMA‐S) Extension states that authors ‘should strongly consider having the search strategy peer reviewed by an experienced searcher, informational specialist, or librarian’ (Rethlefsen et al., 2021). As well, many organisations encourage search peer review (Agency for Healthcare Research & Quality, 2014; Centre for Reviews and Dissemination, 2009; Eunethta, 2019; Institute for Quality and Efficiency in Health Care, 2022; Institute of Medicine, 2011; Lefebvre & Duffy, 2021; National Institute for Health and Care Excellence, 2014; Page, McKenzie, et al., 2021; Page, Moher, et al., 2021; Rethlefsen et al., 2021).

It is recommended that authors provide information on the search strategy development and peer review processes (see Section 1, 9). The PRISMA 2020 explanation and elaboration article (Page, Moher, et al., 2021) and the PRISMA‐S Extension (Rethlefsen et al., 2021) provide guidance on how and where authors should describe the processes used to develop, validate and peer review the search strategy. There is also a CC‐IRMG search peer review checklist, used to evaluate the search strategy during peer review, that review authors can consult to make sure all elements of a search have been included (Appendix IV).

### 6.9 When to stop searching

Developing a search strategy for a database is iterative and involves exploring the impact of search terms on the sensitivity and precision of the search. It is often difficult to decide in a scientific or objective way when a search is complete. The ability to decide when to stop typically develops through experience of developing many strategies.

There are diminishing returns for search efforts; after a certain stage, each additional unit of time invested in searching returns fewer references that are relevant to the review. Consequently, there comes a point where the rewards of further searching may not be worth the effort required to identify the additional references. The decision as to how much to invest in the search process depends on the question a review addresses and the resources that are available. At an estimated reading rate of two abstracts per minute, the results of a database search can be ‘scan‐read’ at the rate of 120 per hour (or approximately 1000 over an 8‐h period), so the high yield and low precision associated with systematic review searching is not as daunting as it might at first appear in comparison with the total time to be invested in the review. Nonetheless, librarians or search experts working on a review should consult with investigators to target the search appropriately within the parameters of the inclusive searching that is conducted for systematic reviews.

Suggestions for stopping rules include stopping if adding new terms yields no new relevant records, if precision falls below a particular cut‐off point (Chilcott et al., 2003), if removal of terms or concepts results in missing relevant records, and checking whether the search is finding the publications that have been recommended as key publications (see Section 5.2) or that have been included in other similar reviews (Cooper, Varley‐Campbell, et al., 2018; Eunethta, 2019). Another consideration is the amount of evidence that has already accrued: in topics where evidence is scarce, authors might need to be more cautious about deciding when to stop searching. Citation searches (see Section 4.2.2) and reference checking (see Section 4.3.2) are useful checks of strategy performance. Statistical techniques, capture‐mark‐recapture (Kastner et al., 2009; Lane et al., 2013), or the relative recall technique (Sampson et al., 2006; Sampson & McGowan, 2011), have also been developed to assess performance.

Unlike reviews of treatment effectiveness where quantitative studies are the primary studies of interest, in evidence synthesis involving qualitative data, searching is often more organic and intertwined with the analysis such that the searching stops when new information ceases to be identified (Booth, 2016). The reasons for stopping need to be documented and it is suggested that explanations or justifications for stopping may centre around saturation (Booth, 2016). Further information on searches for qualitative evidence can be found in the Cochrane Handbook, Chapter 21 (Higgins et al., 2023).

There are no set rules of how much is enough when it comes to grey literature searching. It can be very time consuming to conduct these types of searches. Therefore, it is recommended that the research team discuss where and how to search for grey literature. Consideration should be given to the time and capacity required.

### 6.10 Summary points

- Searches should aim for high sensitivity and review teams should be prepared to accept low precision.
- Searches should be substantively consistent across all databases but tailored for differences in search syntax and controlled vocabulary for individual databases.
- For most Campbell reviews, the search strategy will be comprised of two main concepts: population or condition and intervention. Use of a third concept for outcomes will vary depending on the nature of the question.
- Avoid using too many search concepts in the search strategy. For each concept, use a variety of synonyms and related terms (both natural language keywords and controlled vocabulary terms) combined with ‘OR’ within each concept.
- Combine different concepts with the ‘AND’ operator.
- Avoid use of the ‘NOT’ operator.
- Consider using proximity operators to narrow search retrieval.
- Consider having your search strategy peer‐reviewed.
- The CC‐IRMG peer review checklist (Appendix IV) can be used as a search strategy review tool.

## 7 MANAGING REFERENCES

### 7.1 Introduction

Database searches used to identify studies for systematic reviews tend to retrieve large numbers of references. To organise and work with these references, it is imperative that review teams manage them in an effective and efficient manner. Reference management software serves many purposes but can be used to store and organise references from databases and other sources, identify and remove duplicates, retrieve full text, tag references, and generate in‐text citations along with formatted bibliographies. Of note, other tools described in Section 8.3 may also be used to accomplish similar tasks.

### 7.2 Reference management software

Specially designed bibliographic or reference management software such as EndNote (https://endnote.com/), Zotero (https://www.zotero.org/), or RefWorks (https://refworks.proquest.com/) is helpful and relatively easy to use to manage references found through searches, as well as the full text of studies. Search results may be imported directly into a central database or library, managed (elimination of duplicates, addition of notes, categorisation), and outputted in a variety of citation styles. In addition to the three managers mentioned above, other ones are listed in the Systematic Review Toolbox (http://systematicreviewtools.com/software.php). Some reference management tools are freely available while others require a subscription or a one‐time fee. Some are desktop‐based software that only work on certain operating systems while others are accessible via a web browser. These characteristics along with other features are compared on Wikipedia (https://en.wikipedia.org/wiki/Comparison_of_reference_management_software) and documented in the literature (Gibbs et al., 2022; Meade et al., 2024; Panda, 2023; Saxena & Kaushik, 2022).

When review teams have access to screening software or services such as those described in Section 8.3, reference management software may not be needed. However, there are instances where an RIS export from a database or resource might not be available and where a reference manager's browser extension might allow for certain bibliographic data to be captured. Another example might be when a search tool does not allow for searches to be combined and several separate searches need to be conducted. A reference manager may act as an intermediary to remove duplicates before importing those references into a screening tool. Some reference managers can also be used to find full‐text articles in batches.

For review teams that do not have access to screening software or services, reference managers may also be used to review, screen, and select studies (Bramer et al., 2017; Mendes et al., 2019).

### 7.3 Removing duplicates

To identify studies, review teams will search multiple databases to ensure their search is comprehensive. Doing so will inevitably lead to finding the same reference more than once. It is imperative that review teams find an approach for removing duplicates efficiently to avoid extra work in screening the same reference more than once.

Screening tools described in Section 8.3 often remove duplicates when RIS files are imported. With reference managers, de‐duplication features may vary. Some identify duplicates automatically while others require manual intervention.

De‐duplication is fraught with potential issues and no single solution is foolproof. When automated, the process may be prone to identify false positives by removing references that are actually unique as well as false negatives by keeping references that are actually duplicates. This happens because bibliographic data for the same study may vary across databases, and algorithms used to identify duplicates may not capture these nuances. When done manually, identifying duplicates is extremely time‐consuming.

Review teams may turn to published studies for guidance on best practices for removing duplicates such as considering free tools (Guimarães et al., 2022), comparing reference managers with screening tools (McKeown & Mir, 2021) or turning to newer de‐duplication algorithms (Borissov et al., 2022). Because the number of duplicates removed across sources will need to be reported in the PRISMA flow diagram (Page, McKenzie, et al., 2021), it is imperative that this process be well thought out and documented.

### 7.4 Summary points

- Manage references effectively through the review process by relying on reference management or screening software or services.
- Use these tools to store, organise, tag, screen, export and cite references.
- Consider important advantages and limitations of using these tools to remove duplicate records.

## 8 SELECTING STUDIES

### 8.1 A typical process for selecting studies

A typical process for selecting studies may include the following steps.

- Merging of records obtained from various database searches to remove duplicate records (identical records that have the same metadata such as title, authors, journal, year, etc.).
- Screening of records, based on titles and abstracts only, to remove clearly irrelevant records. It is recommended that authors err on the side of inclusion at this stage, in order to allow for evaluation of the full text. This stage may be implemented in three steps: piloting the process/criteria, screening of all title‐abstracts, resolving discrepancies in the screening decisions.
- Retrieval of the full text (PDFs or HTML) of each record that was included from the title‐abstract screening stage.
- Evaluation of full‐text reports against the eligibility criteria of the review to decide on inclusion or exclusion, with reason for exclusion clearly documented.

Multiple reports of the same study may exist. Authors should not discard multiple reports of the same study in case they contain additional details of the study design and conduct. Authors may merge these reports either before or after full‐text evaluation. In other words, the unit of interest in a systematic review is a ‘study’ and not a ‘publication’ (of which there may be multiple for a given ‘study’).

Furthermore, authors may need to contact the corresponding author of reports of studies to confirm or clarify study details, if unclear in the available text, before making the final decisions.

The planned process, including specific references to process, persons involved, and software should be outlined in the protocol.

### 8.2 Implementation of the selection process

Once the search has been conducted and duplicate records removed, screening for eligible studies can commence. Decisions about eligibility will be made based on predetermined criteria for inclusion or exclusion established by the review authors during the protocol development stage. Screening usually happens in two steps: title/abstract screening followed by a review of full‐text; however, teams may decide to conduct title‐only screening as a first step prior to looking at abstracts.

Screening should be conducted with each record being reviewed by at least two independent reviewers (MECCIR Standard 4, https://doi.org/10.17605/OSF.IO/KCSPX) at each stage. It is highly recommended that the criteria be piloted on a set of studies. In this way, disagreements and misunderstandings about the criteria and concept definitions can be resolved before too many records have been processed. The aim is to have as clear criteria as possible to minimise the number of conflicting decisions between reviewers and ultimately reduce the risk of bias in record selection. Review teams typically develop additional guidance on applying the criteria, which can be updated for clarity and consistency through the screening process. Substantive changes that deviate from the protocol should be avoided throughout the review itself. Changes made during the review should be reported transparently in the final manuscript such that deviations from the protocol are clear.

The team may consider calculating the interrater reliability (IRR) of reviewers during the pilot and using a cutoff (e.g., 75% or 80%) (Aromataris et al., 2024; McHugh, 2012) to establish a transparent metric for concluding the pilot (Belur et al., 2021). This piloting can happen both at the title/abstract and full‐text screening stages. For the title/abstract screening pilot, typically between 25 and 100 studies, randomly chosen from the results set, can be screened (Aromataris et al., 2024). More may need to be screened if the IRR is low. For the full‐text screening pilot the number of studies is usually lower and will depend on how many studies are included at the full‐test screening stage.

During title/abstract screening reviewers choose whether to move a study into the full‐text screening phase. Only studies that can clearly be excluded based on the title and abstract alone are removed at this stage – this includes studies failing to meet one or more of the eligibility criteria. Studies that meet eligibility criteria or for which there is insufficient information in the title and abstract to decide are moved onto the full‐text screening phase. When reviewers make conflicting decisions, these conflicts are resolved by a third reviewer or by discussion and consensus between reviewers. At the title/abstract stage, reasons for exclusion are not typically recorded. In mixed methods reviews, when screening for qualitative studies Skalidou and Oya (2018) describe a need to be flexible and inclusive to allow for variations in reporting.

The studies that move onto the full‐text screening stage will be reviewed in full to make a final determination about their eligibility. Full‐text articles can be found via library collections, open access journals or other sources where available, including interlibrary loan services provided by authors' institutional libraries. Studies for which the full text is not available after exhaustive attempts are noted as unretrievable. The total number of unretrieved records is reported in the PRISMA flow diagram (Page, McKenzie, et al., 2021). For retrieved articles, two independent reviewers make determinations about eligibility and as above, conflicts are resolved by a third reviewer or by discussion and consensus. For the full‐text screening stage, reasons for exclusion are recorded and must also be agreed upon by reviewers. Reasons for exclusion should mirror the predefined eligibility criteria. Only one reason for exclusion can be recorded per article even if the article meets multiple exclusion criteria. It is therefore recommended that exclusion reasons be ordered (in the form of a hierarchy of exclusion reasons) based on importance or ease of determination, such that the first criteria not met by a record would be used and noted as the exclusion reason. It can be helpful to calculate and report the IRR (e.g., using Cohen's Kappa; simple percentage [number of records with agreement between reviewers/total number of records reviewed by those reviewers]) of the screening stages to help readers better understand the risk of bias in selection of included studies (Belur et al., 2021).

### 8.3 Software support for selecting studies

Because the searches in systematic reviews often result in thousands of records, the screening process can be labour‐intensive and logistically challenging. For this reason, it is highly recommended that teams use specialised software for managing the screening process. These softwares can also facilitate the documentation of included and excluded studies, which is critical for proper reporting and the development of the PRISMA flow diagram. It is now common for these tools to incorporate machine learning and other tools/techniques that can increase speed and efficiency of the relevance screening phase.

#### 8.3.1 Software for managing the selection process

There are numerous software tools available for screening studies in systematic reviews. Many of these can be found in the Systematic Review Toolbox (http://systematicreviewtools.com), which is a web‐based catalogue of tools supporting different evidence synthesis methods and steps (Marshall & Brereton, 2015). Some of the considerations when choosing a screening software include:

- Cost and availability: Many screening tools are free or provide a free version, while others require a subscription or one‐time fee. At the time of publication of this guide, Campbell provides free access to Covidence (https://www.covidence.org/) and EPPI‐Reviewer (https://eppi.ioe.ac.uk/eppireviewer-web/home). These tools can support screening, data extraction and analysis. Some institutional libraries may provide subscriptions to tools like Covidence or DistillerSR (https://www.distillersr.com/products/distillersr-systematic-review-software).
- Functionality: Screening platforms differ in the steps supported. For example, some tools can be used to deduplicate records, others may provide support for later phases of the review like data extraction, and some automatically produce output required by common reporting guidelines, such as the PRISMA flow diagram and IRR.
- Machine learning applications: Increasingly, machine learning techniques can be applied to facilitate or speed up the screening process. This is typically implemented in the form of an algorithm trained by reviewer decisions such that records are resorted or assigned a prediction score for eligibility based on previous decisions already made by screeners (O'Mara‐Eves et al., 2015). It is intended that records that are more likely to be relevant are presented to the reviewers earlier in the review.

In most cases and at a minimum, screening software designed for systematic reviews should allow for the uploading of records exported from databases (often in RIS format), provide a view of titles and abstracts, and provide methods for two or more collaborators to make Yes/No decisions about eligibility. Ideally, screening platforms should also allow for the uploading of full text articles and for customising eligibility criteria.

### 8.4 Summary points

- Merge records and remove duplicates.
- Select a review management software to help with the screening process.
- Pilot the eligibility criteria before screening all records.
- Screen record titles and abstracts based on eligibility criteria.
- Retrieve the full text of each record.
- Evaluate full‐text reports against eligibility criteria.

## 9 DOCUMENTING AND REPORTING THE SEARCH

One of the defining characteristics of systematic reviews in contrast to traditional literature reviews is the transparent reporting of methods such that the review could be replicated. This level of transparency requires thorough documentation throughout the review process itself. As the foundation of the review, the search must also be reported transparently such that the search is reproducible.

It is recommended that review authors work with an IS or librarian (see Section 2) at the earliest opportunity to ensure proper documentation of the search process.

### 9.1 Reporting guidelines

The search process needs to be documented in enough detail to ensure that it can be reported in the final manuscript such that all the searches of all databases are reproducible. It is helpful to become familiar with the requirements of relevant reporting guidelines at the start of the review to ensure the team documents all the required reporting items throughout the search process.

#### 9.1.1 MECCIR

Those conducting a Campbell review are required to adhere to the MECCIR standards (https://doi.org/10.17605/OSF.IO/KCSPX) at a minimum. Please note that the current version of MECCIR requires all items to be addressed in both the protocol and final manuscript.

According to MECCIR Item 3 Search Strategy:

The goal of the search is to identify all eligible studies and to do so in a way that is transparent, replicable, and reduces the potential to further publication bias. To do this one needs to search multiple databases as well as grey literature and the reference lists of existing reviews and included studies. Additional methods, such as conducting a forward citation search of seminal works and included studies and hand searching key journals, can be used to improve comprehensiveness. The searches of databases should involve a well‐thought‐out set of keywords and make effective use of Boolean logic, wild cards, phrase searching, and subject headings, although one should be cautious of using some built‐in database filters. If possible, involve a librarian or information retrieval specialist with systematic review experience. A careful log of the search should be maintained and reported in the final review, including when the search was conducted, the results of the search, database and platform names, and the exact search strategy for each database. This includes all keywords, subject headings, and database syntax used for each bibliographic database and for grey literature resources when applicable. It is important to search for unpublished studies to mitigate the influence of reporting bias, including contacting experts in the field.

Regarding conduct, MECCIR Item 3 requires review teams to:

Develop a search strategy that has a reasonable probability of identifying all studies that match eligibility criteria.

#### 9.1.2 PRISMA‐S

Although not required for Campbell systematic reviews, Campbell review teams are strongly encouraged to adhere to the PRISMA‐S reporting guidelines which establish more detailed standards for reporting the search than MECCIR. PRISMA‐S is an extension to the PRISMA reporting guidelines and was designed to be used in all fields and disciplines (Rethlefsen et al., 2021).

### 9.2 Documenting the search process

The information that your team documents and keeps track of during the search process should be informed by MECCIR and PRISMA‐S reporting standards/guidelines (see Section 9.1). A well‐documented search will facilitate writing this section of the review manuscript.

The search strategies for each database will need to be copied and pasted exactly as run and included in full, together with the total number of search results and the number of records downloaded. The number of records retrieved from each database search will need to be recorded in the Appendix. The search strategies should not be re‐typed as this can introduce errors. Many databases offer an option to print, email, or download the search history which should be used where available. See Appendix III for a template that may be used for this purpose.

#### 9.2.1 Reporting the search process in the protocol

The MECCIR standard should be used to guide the development of a Campbell review protocol. The protocol should describe all of the items you would be expected to report in the final manuscript (see Section 9.1.2) but will be phrased as actions the team will take (future tense) rather than what has already been done (past tense). It is likely that some items cannot be fully addressed in the protocol if final searches have not been conducted. Teams may also consider using the PRISMA‐P (Moher et al., 2015) extension for reporting systematic review protocols. Item 10 in PRISMA‐P relates to the search strategy and is described with the following checklist statement:

Present draft of search strategy to be used for at least one electronic database, including planned limits, such that it could be repeated.

#### 9.2.2 Reporting the search process in the final review manuscript

The following data elements, described by MECCIR and PRISMA‐S, respectively, should be reported for the search strategy to be considered transparent and increase the likelihood of being reproducible.

Item 3 from MECCIR Standards:

‘Describe search strategy in enough detail for replication’ (search terms for electronic bibliographic databases, grey literature, registries, forward citations, etc.)

Reporting Items from PRISMA‐S

- Information about sources:
  o Name of each database and platform (interface) through which each database was searched.
  o If applicable:
    ▪ Whether multiple databases were searched simultaneously.
    ▪ Study registries searched.
    ▪ Online or print resources handsearched.
    ▪ whether citation searching was conducted and, if so, approach used.
    ▪ Whether relevant individuals or groups were contacted for additional studies.
    ▪ Additional methods used to find studies.
- Search strategy details:
  o Full search strategy for each database (including all database syntax as run in each database) and, if applicable, additional information sources.
  o Limits applied to the search and justification for use; if no limits, indicate no limits were applied.
  o Approaches to updating the corpus (e.g., setting search alerts, rerunning the search) prior to publication.
  o Dates of final search in each database.
  o If applicable:
    ▪ Search filters used, whether in their original form or modified; if used, cite (see Chapter 6.5.5 for more information about search filters).
    ▪ Other resources used to develop the search strategy (e.g., existing reviews).
    ▪ Date of search rerun for updating the corpus.
- Peer review:
  o Process for peer review of the search strategy.
- Management of records:
  o Total search yield from each database.
  o Process for deduplicating results; software used, if applicable.

##### Reporting additional search strategies

Evidence from sources other than academic databases may be particularly important for Campbell reviews (e.g., searching websites, contacting individuals or groups). PRISMA‐S provides specific guidance for each of the items expressly mentioned in Section 9.1.2, above. For example, for reporting contact with authors (PRISMA‐S Item 6), they suggest ‘As these strategies are inherently difficult to reproduce, researchers should attempt to give as much detail as possible on what data or information was sought, who or what group(s) provided data or information, and how the individuals or groups were identified’ (Rethlefsen et al., 2021). As PRISMA‐S notes, because of the inherent variability in these processes, it is unlikely that these approaches will be replicable.

For website searches, the following items should be documented (Stansfield et al., 2016). Those in bold should be reported (Briscoe, 2018) (see Appendix III for an example template):

- **Name of Resource**
- **URL**
- **Date Searched**
- **Search Strategy** (includes all aspects relating to the search process, such as search terms and search limits (e.g., date limits), and descriptive detail such as sections of websites searched).
- ‘Pathway followed, e.g., Browsed headings/searched site/database within website (use separate lines for the different types of searches)’.
- Whether predefined keywords from a website menu or filter were used.
- Any notes that may help communicate the search process transparently to the team and/or support reporting.
- Name of person who ran the search.

In many reviews, additional searches in sources other than bibliographic databases are important to include for the sake of comprehensiveness. For these additional searches, review teams are generally advised to document and report their approach with as much detail as possible, aiming for a level of transparency that will allow the reader of their review to be able to assess whether these approaches are sufficiently reliable for their use of review findings.

As mentioned elsewhere in this document, it is important to save copies of any information found on the internet as this information may no longer be accessible at the time the review is written. Whenever possible, record persistent links (permalinks) and/or digital object identifiers (DOIs) rather than session links which will expire.

### 9.3 Summary points

- Refer to the MECCIR standards, and ideally the PRSIMA‐S reporting guideline early and often.
- Throughout search development, document thoroughly.
- Seek guidance for documenting the search process from an IS or librarian before starting the search.
- Copy and paste (or print, download, etc.) the full strategy for searches of all sources into the review.
- Use the example templates to help document this process (Appendix III).
- Document and report additional search approaches (e.g., for grey literature) with enough detail that the reader can evaluate the reliability of review findings.
- Save locally or file print copies of any information found on the internet; use persistent links, DOIs, and so forth, rather than session links.
